# A universal karyotypic system for hexaploid and diploid *Avena* species brings oat cytogenetics into the genomics era

**DOI:** 10.1186/s12870-021-02999-3

**Published:** 2021-05-12

**Authors:** Wenxi Jiang, Chengzhi Jiang, Weiguang Yuan, Meijun Zhang, Zijie Fang, Yang Li, Guangrong Li, Juqing Jia, Zujun Yang

**Affiliations:** 1 Center for Informational Biology, School of Life Science and Technology, University of Electronic Science and Technology of China, 611731 Chengdu, China; 2 College of Agronomy, Shanxi Agricultural University, 030801 Taigu, China

**Keywords:** *Avena*, Oligo-probes, Chromosome identification, FISH

## Abstract

**Background:**

The identification of chromosomes among *Avena* species have been studied by C-banding and *in situ* hybridization. However, the complicated results from several cytogenetic nomenclatures for identifying oat chromosomes are often contradictory. A universal karyotyping nomenclature system for precise chromosome identification and comparative evolutionary studies would be essential for genus *Avena* based on the recently released genome sequences of hexaploid and diploid *Avena* species.

**Results:**

Tandem repetitive sequences were predicted and physically located on chromosomal regions of the released *Avena sativa* OT3098 genome assembly v1. Eight new oligonucleotide (oligo) probes for sequential fluorescence *in situ* hybridization (FISH) were designed and then applied for chromosome karyotyping on mitotic metaphase spreads of *A. brevis*, *A. nuda*, *A. wiestii*, *A. ventricosa*, *A. fatua*, and *A. sativa* species. We established a high-resolution standard karyotype of *A. sativa* based on the distinct FISH signals of multiple oligo probes. FISH painting with bulked oligos, based on wheat-barley collinear regions, was used to validate the linkage group assignment for individual *A. sativa* chromosomes. We integrated our new Oligo-FISH based karyotype system with earlier karyotype nomenclatures through sequential C-banding and FISH methods, then subsequently determined the precise breakage points of some chromosome translocations in *A. sativa*.

**Conclusions:**

This new universal chromosome identification system will be a powerful tool for describing the genetic diversity, chromosomal rearrangements and evolutionary relationships among *Avena* species by comparative cytogenetic and genomic approaches.

**Supplementary Information:**

The online version contains supplementary material available at 10.1186/s12870-021-02999-3.

## Background

The common oat (*Avena sativa* L., 2n = 6x = 42, AACCDD) is a temperate crop (annual production of 23 million tons in 2017; http://faostat.fao.org) which is primarily used for livestock feed and partially for human food. It is also a food crop recommended by nutritionists because its consumption helps reduce blood cholesterol levels and heart disease risk [1, 2]. The genus *Avena* consists of several diploid, tetraploid and hexaploid species. All diploid species contain either the AA or CC genomes, the tetraploid species carry the AABB, AACC, CCCC or CCDD genomes, while the hexaploid species have AACCDD genomes [3]. The cultivated oat was domesticated from the wild and weedy *A. sterilis* L., which arose from hybridization between a CCDD allotetraploid and an AsAs diploid [4, 5].

*Avena* represents a remarkable model to study because of its history of polyploidy, lineage divergence and complex reticulate evolution [6–8]. The relationships and origins among *Avena* species have been intensively studied by molecular and cytological approaches [8, 9]. The earliest investigations into genome structure involved meiotic chromosome pairing, C-banding and genomic *in situ* hybridization (GISH) which enabled the sub-genome chromosomes in common oat to be distinguished [6–13]. Identification of the chromosome complements of *Avena* species has been successfully achieved using fluorescence *in situ* hybridization (FISH) based on the satellite sequence pAs120a, specific to the A genome, in combination with rDNA probes and the sequence pAm1, satellite DNA specific to the C genome [14, 15]. Several cytogenetic nomenclatures have been established for identifying all 21 hexaploid oat chromosomes and assigning these chromosomes to each of the three subgenomes [16–18]. However, the results from different researchers are often complicated and require specialized training to interpret and reproduce due to a lack of conserved FISH probes and frequent intergenomic rearrangements which have occurred in the different oat species [19, 20]. Establishment of a universal karyotyping nomenclature system for each individual oat chromosome pair would be of enormous benefit to *Avena* researchers.

FISH using probes from different families of satellite sequences can generate chromosome- and genome-specific patterns, and consequently allows for the identification of chromosome pairs at mitotic metaphases [21, 22]. The non-denaturing fluorescence *in situ* hybridization (ND-FISH), using oligonucleotide (oligo) probes of repetitive sequences, has been confirmed as a simple, cheap and high-throughput method for painting the chromosomes of different plant species [23–26]. The FISH signal patterns of some probes, including simple sequence repeat (SSR) motifs, have previously been documented for several different oat lines [20, 27–31]. However, the FISH patterns of those SSRs are highly polymorphic, which may cause difficulties for the assignment of specific chromosomes to certain sub-genomes and linkage groups.

Genome research on oat has received less attention than for wheat (*Triticum aestivum* L.) and barley (*Hordeum vulgare* L.), possibly because it is not a relatively prominent component of the human diet [32–34]. Maughan et al. [35] reported two complete genome sequences of representative diploid *Avena* spp., *A. atlantica* (A genome) and *A. eriantha* (C genome), and the first hexaploid oat OT3098 reference genome v1 has also been released recently (https://wheat.pw.usda.gov/GG3/node/922). These genome databases have enabled researchers to employ bioinformatic approaches to determine the genomic locations of tandem repeats (TR) along the oat chromosomes [25, 36, 37] and develop FISH probes suitable for chromosome identification. Moreover, oligo-FISH painting systems using the bulked pools of 40–50 bp lengths, which are specific to an entire chromosome or a specific region, have successfully enabled the karyotyping of several sequenced plant species with various genome sizes [22, 37–39]. The oligo-FISH painting system was recently used to assign specific linkage groups to chromosomes in Triticeae species [37]. Therefore, this comparative oligo-FISH painting system may also be modified to enable karyotyping of the oat chromosomes, since the divergence time between oats and members of the Triticeae tribe has been estimated to be only 25.5–26.5 MYA [40].

In the current study, we predict the chromosomal distribution of tandem repeats (TRs) by use of a web server analyzing data from recently released *Avena* genome sequences. The TR-oligo based ND-FISH and single copy oligo pool-based FISH painting methods have been integrated to establish a standard nomenclature system for identification of individual chromosomes of *Avena* species. Our study will fill the important gap in the cytogenetic and genomic approaches for precise identification of chromosome segments and rearrangements for future oat improvement.

## Results

### Genomic distribution of TRs in *A. sativa* genomes

In order to investigate the proportional contribution of the TRs in *A. sativa* genomes, the sequence data of all 21 individual chromosomes from the cultivated oat OT3098 reference assembly v1 were analyzed [25]. We obtained 10,832 arrays of TRs comprising a total length of 447.21 Mb, which constitutes 4.16 % of the total 10.74 Gb assembled oat genome (Table S1). The lowest proportion of chromosome sequence length contributed by TRs was for chromosome 6D at 1.11 %, and highest proportion of sequence length contributed by TRs was for chromosome 6 C reaching 8.67 % (Figure S1a). The distribution of TRs across the seven different linkage groups was also compared, and a clear variation was observed (Figure S1b). The coverage of TR content varied across the A, C and D genomes. For both the overall length and the percentage of TRs of the chromosomes for the C genome were much higher than for the A and D genomes. The average TR content among the A, C and D genomes was 2.59 %, 6.92 and 2.17 %, respectively (Figure S1c).

### Physical locations of known TRs and validation by ND-FISH

**Fig. 1:**
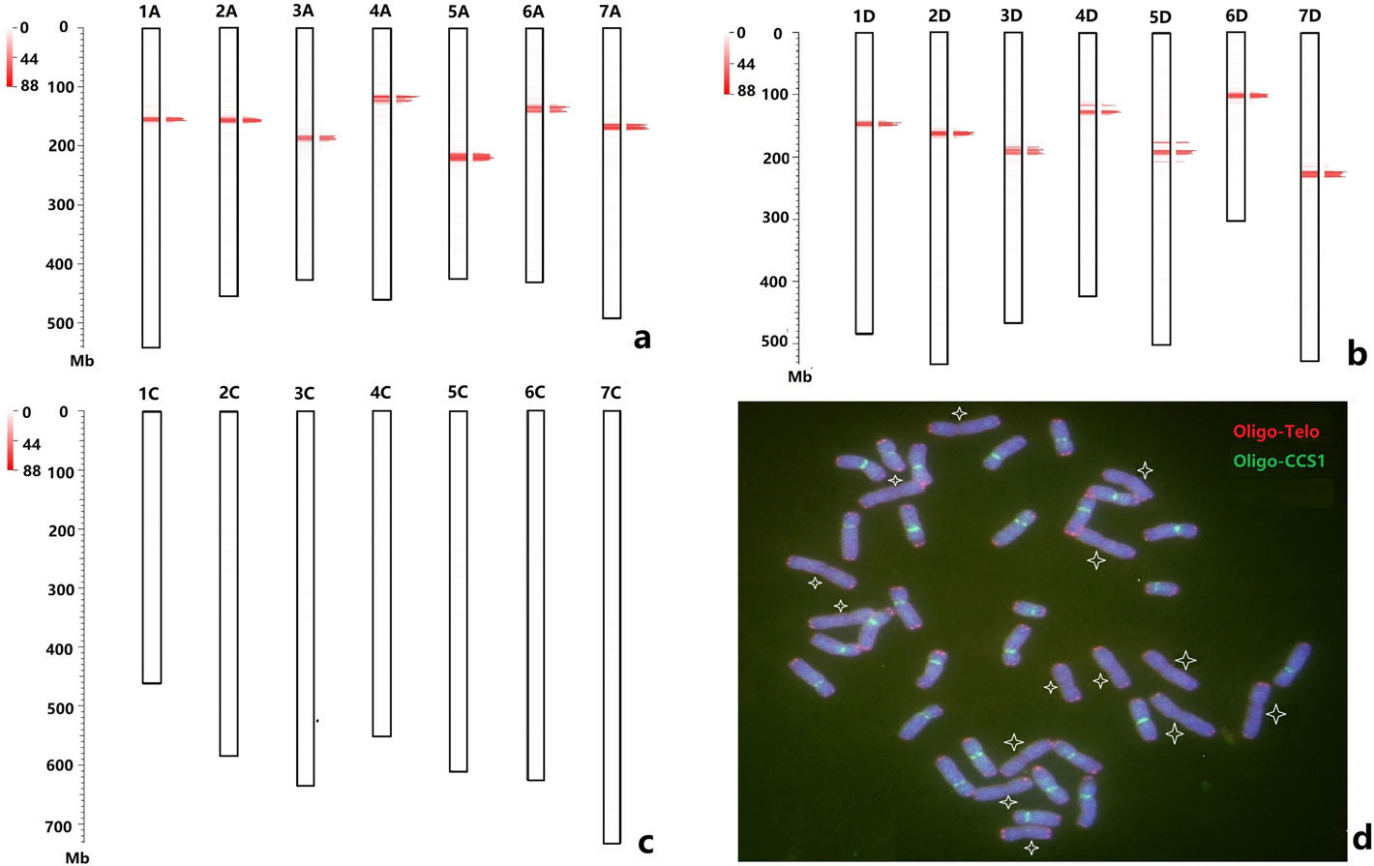
The physical distribution (**a**-**c**) and ND-FISH results (**d**) of Oligo-CCS1 in chromosomes of oat. **a**-**c** The prediction of Oligo-CCS1 in oat OT3098 reference assembly v1 was according to website B2DSC by using default parameters for the blast and filter steps. The parameters pident = 85, qcovhsp = 80. Red bars: the positions of Oligo-CCS1. **d** ND-FISH for mitotic metaphase of BaiyanII showing the hybridization of Oligo-CCS1 (green) + Oligo-Telo (red) on 28 chromosomes, with the 14 chromosomes of C-genome devoid of centromeric hybridization signals (star marked)

FISH probes, usually several hundred base pairs in length, have been previously used for identification of mitotic metaphase chromosomes of *Avena* species [41–44]. A conserved repetitive DNA element was located in the centromeres of cereal chromosomes, and subsequently was also localized to centromeric regions of oat chromosomes [45, 46]. The predicted copy number of the cereal centromeric sequence (CCS1) for *A. sativa* was estimated by the D2DSC web server [25], and the distribution on oat chromosome regions is shown in Table S2. We found that this CCS1 repeat was distributed mainly on the centromeric regions of chromosomes of the A and D genomes (Fig. 1a-c). ND-FISH using probe Oligo-CCS1 showed clear centromeric signals on 28 chromosomes, which are presumably the oat A and D genomes, but lacked any hybridization on the C genome of BaiyanII (Fig. 1d). This result is consistent with the FISH study of Tomas et al. [19]. We thus propose that the predicted locations of Oligo-CCS1 sites match with the cytological positions of the centromeres, suggesting the precision and validity of the sequence assembly for *A. sativa*.

**Fig. 2:**
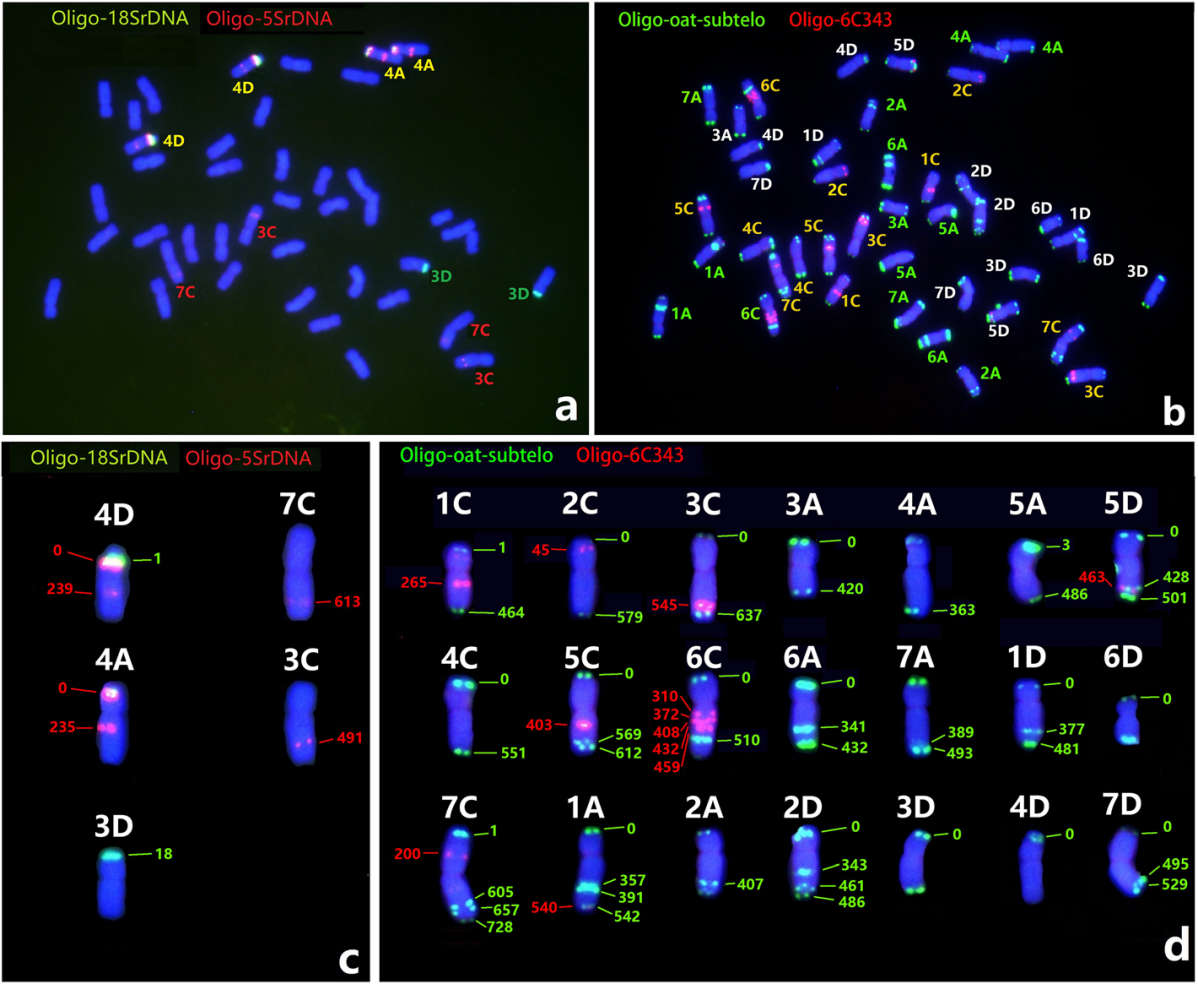
The ND-FISH of oligo probes Oligo-5SrDNA + Oligo-18SrDNA (**a**) and Oligo-oat-subtelo + Oligo-6C343 (**b**), and their hybridization sites (**c**, **d**) on metaphase chromosomes of *Avena sativa* cv. BaiyanII The numbers (in Mb) show the central physical positions predicted on the *A. sativa* reference genome assembly v1 using the B2DSC web server

The relative positions of the 5 and 18 S rDNA loci is a highly conserved characteristic of cereal and oat genomes [47, 48]. The distribution of 12 major 5 S rDNA signal sites, located on four oat chromosome pair, was revealed by ND-FISH probed by Oligo-5SrDNA (Fig. 2). The predicted physical locations of 5SrDNA, based on the oat reference genome v1, are shown on the assembled oat chromosomes 4 A, 4D, 3C and 7 C (Fig. 2c), and these sites are consistent with the FISH results. Similarly, studies were also conducted on the predicted physical locations and subsequent FISH verification of 18SrDNA on oat chromosomes 4 A and 3D (Fig. 2a and c).

**Fig. 3:**
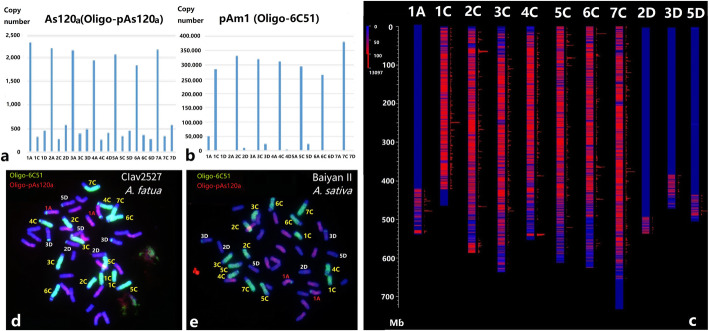
Copy number prediction and FISH validation of Oligo-pAs120a and Oligo-6C51 on hexaploid oat chromosomes. The copy number prediction of As120a (**a**) and pAm1 (**b**) was shown on *A. sativa* genome assembly v1, and a visualized distribution of pAm1 (Oligo-6C51) was indicated (**c**) by B2DSC web server. The probes Oligo-pAs120a + Oligo-6C51 (**d**, **e**) were used for FISH of lines Clav2527 (*A. fatua*) and BaiyanII (*A. sativa*), respectively

Linares et al. [49] isolated a 670-bp satellite DNA sequence, As120a, (NCBI genbank number: AJ001922) specific to the A-genome chromosomes in *A. sativa*. An oligo probe Oligo-pAs120a was designed based on the As120a sequences (Table 1). The physical distributions and copy number estimation of Oligo-pAs120a, predicted by B2DSC for the *A. sativa* genome assembly v1, are shown in Fig. 3. The results showed that As120a displays about 1,800 to 2,300 copies for each A-genome chromosome, while the C and D genomes have fewer than 600 copies per chromosome. FISH with probe Oligo-pAs120a on cultivated oat mitotic spreads revealed that 14 chromosomes showed clear hybridization, and thus we conclude that these are the A- subgenome chromosomes (Fig. 3e).

Solano et al. [50] reported a satellite DNA sequence, pAm1, specific to the oat C genome, containing an insert of 464 bp (NCBI genbank number: X83958.1) isolated from *A. murphyi*. Based on the tandem repeats prediction in the *A. sativa* genome v1 by the B2DSC web server, we found that pAm1 repeats contained a core consensus 51 bp monomer sequence with a copy number of around 260,000-380,000 among the C-genome chromosomes (Fig. 3b). A probe named Oligo-6C51 (Table 1), representing pAm1 for ND-FISH, was hybridized to the metaphase cells of *A. sativa* and *A. fatua*. As shown in Fig. 3d and e, the probe Oligo-6C51 had extremely strong hybridization signal sites located across the entire 14 C genome chromosomes of oat cultivar BaiyanII and *A. fatua* Clav2527. Faint signals also appeared on the telomeric regions of four chromosome pairs (Fig. 3d, e), which are consistent to the predicted chromosomes 1 A, 2D, 3D, and 5D (Fig. 3c). Therefore, the ND-FISH hybridization patterns of probes Oligo-6C51 for the C genome and Oligo-pAs120a for the A genome will facilitate sub-genome assignment of individual chromosomes in the hexaploid oat, with the remaining D-genome chromosomes showing limited hybridization by these two probes.

### Production of new repetitive probes for chromosome identification

**Fig. 4:**
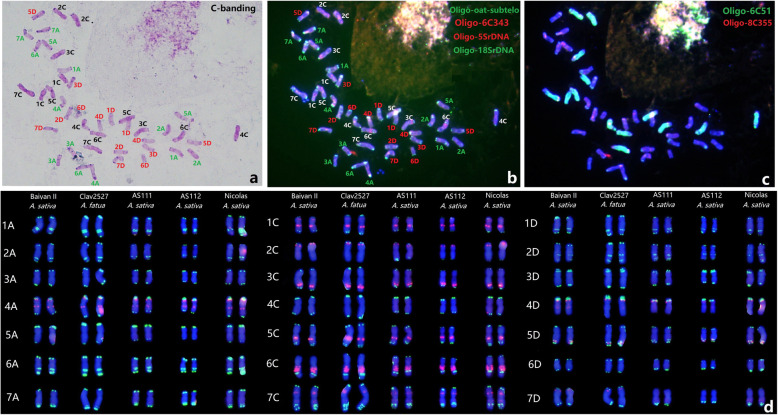
The sequential C-banding (a) and FISH (b-d) for karyotyping hexaploid oat. The metaphase cell of BaiyanII was revealed by C-banding (**a**) and sequential FISH with the probes Oligo-oat-subtelo + Oligo-6C343 + Oligo-18SrDNA + Oligo-5SrDNA (**b**), Oligo-6C51 + Oligo-8C355 (**c**), respectively. The karyotype diagram of Oligo-oat-subtelo + Oligo-6C343 for BaiyanII, Clav2527, AS111, AS112 and Nicolas were indicated (**d**)

In order to establish the standard karyotype of hexaploid oat, we need to produce more probes with each of their physical positions clearly defined for precise chromosomal painting. In this present study, a total of eight novel oligo probes were designed from the predicted TR database (Table 1), then their physical distributions and estimation of copy numbers were obtained using the B2DSC web server. These oligos appeared as distinct and stable hybridization signals on the chromosomes of BaiyanII by ND-FISH. As shown in Fig. 2, the probe Oligo-oat-subtelo produced hybridization signals on telomeric or sub-telomeric regions of one or both arms of almost all chromosomes, while Oligo-6C343 produced hybridization signals on chromosomes 1 C, 2 C, 3 C, 5 C, 6 and 7 C (Fig. 2). The oligo-probe Oligo-4A70 had hybridization sites only on the distal region of the short arm of chromosome 4 A. Therefore, in combination with A and C- genome specific probes, the oat chromosomes were also recognizable by using a cocktail of the probes Oligo-oat-subtelo, Oligo-6C343 and Oligo-8C355 (Figs. 3 and 4). The hybridization patterns of above mentioned probes for individual oat chromosomes, and their physical locations with units of Mb in the *A. sativa* genome, will be able to distinguish all oat chromosomes with different origins (Figs. 2, 3 and 4). As expected, some probes were successfully hybridized to chromosomes by ND-FISH, but their copy number by TR prediction was largely under-estimated, which possibly implies that the sequence assembly v1 of the related region needs to be improved.

**Table 1:** The sequences of TRs oligo probes for *Avena* chromosome identification by ND-FISH

Oligo probes	Sequences	Reference
Oligo-CCS1	CCGTTTGATAGAGGCAAAGGTGTCCCGTCTTTTGATGAGA	[23]
Oligo-oat-subtelo	CAAACATGTATCGGGTCTTACGGTCATTTTAAATCGCCCT	This study
Oligo-6C51	AACACACATGCAATGACTCTAGTGGTTGATCCATGTGTGGTTTGTGGAAAG	This study
Oligo-6C343	AGGACATATGTACATGGAGAGCCAAGGTTGGGCCAACTTTGCCACATTCT	This study
Oligo-8C355	ACTTTCTTCTGACAGGGGTAGCCCGGTGTAGCCCTCACTTGTTTTA	This study
Oligo-4C709	ATGTGATGATGTAAAACCATGTTTGGGAACATGTTGTGACAAGATCTAC	This study
Oligo-3A352	GTGCTTGCATGTGTCCCCCCTCGCATGCATGCGCTCTAACCTAGAGGCGAA	This study
Oligo-6C686	GAGCCAAGGTTGGGCCAACTTTGCCACATTCTAGGCCCCGGTTGTGACGCGGCGG	This study
Oligo-4A70	AACACTTTCAAATTAAAAAAATTATACAACTCTTAATGTAAAAGAGTGT	This study
Oligo-pAs120a	GGTTTATCTCATACTATCTGTACCTGATTAGTAATTGTTGTAACTACAACGGAATGGTTAACT	[49]
Oligo-18SrDNA	GGGCAAGTCTGGTGCCAGCAGCCGCGGT	[25]
Oligo-5SrDNA	GTACTACTCTCGCCCAAGCACGCTTAACTTCGGAGTTCTGA	[25]
Oligo-HvCSR	ACAACGACAACAACGACAATGACGAGA	[25]
Oligo-Telo	TTTAGGGTTTAGGGTTTAGGG	[24]
Oligo-(CAA)_7_	CAACAACAACAACAACAACAA	[24]
Oligo-(GAA)_7_	GAAGAAGAAGAAGAAGAAGAA	[24]
Oligo-(TAG)_7_	TAGTAGTAGTAGTAGTAGTAG	[24]
Oligo-(ACT)_6_	ACTACTACTACTACTACT	[24]
Oligo-(GT)_10_	GTGTGTGTGTGTGTGTGTGT	[24]

### Chromosome nomenclature system comparison

Jellen et al. [10] reported a karyotyping system based on C-banding analyses of *A. sativa*. Sanz et al. [17] proposed a FISH karyotype of the 21 chromosome pairs of *A. sativa* deduced from analyses using rDNA probes and satellite sequences specific to either the A or C-genome chromosomes. In the present study, we carried out the sequential C-banding and ND-FISH for the first time to the same metaphase cells of *A. sativa* BaiyanII (Fig. 4). The C-banding technique used here demonstrated that the C-genome chromosomes displayed strong staining because of accumulated heterochromatin (Fig. 4a). The specific distribution of C-bands enabled most of the A and D genomes chromosomes to be clearly distinguished (Fig. 4a). The identical cell was subsequently confirm chromosome identification by ND-FISH using the above mentioned probes, including the oligo combinations of Oligo-oat-subtelo + Oligo-6C343 + Oligo-18SrDNA + Oligo-5SrDNA (Fig. 4b) and Oligo-6C51 + Oligo-8C355 (Fig. 4c). The results of C-banding and FISH patterns enabled us to combine the ND-FISH chromosome nomenclature for *A. sativa* used in the present study with the C-banding-based designations of Jellen et al. [10], and the FISH mapping study of Sanz et al. [17]. As shown in Table 2, the new nomenclature system allows numbering of all of the 21 chromosome pairs of *A. sativa* based on genome assembly v1.

Our chromosome identification system, based on ND-FISH with multiple probes, was used to develop karyotypes of the *A. sativa* lines BaiyanII, Clav2527, AS111, AS112 and Nicolas (Fig. 4). The proposed karyotype of hexaploid oat is also shown in Figure S2. We found that all five lines displayed identical karyotypes without any obvious translocations revealed by ND-FISH. Therefore, this new, uniform nomenclature system should be useful in oat cytogenetics, facilitating the identification of homeologous relationships among the chromosomes of the three sub-genomes in *A. sativa*.

**Table 2:** Comparative karyotype nomenclature system of the present genomic based system to those by C-banding, FISH analysis for *A. sativa*

Genomes	Genome based chromosomes ^a^	SSR based FISH (Linares et al. [49])	C-banding (Jellen et al. [10])	FISH and banding (Sanz et al. [17])
A	**1 A**	5 A	17	17 A
	**2 A**	8 A	8	8 A
	**3 A**	18 A	15	15 A
	**4 A**	12 A	19	19 A
	**5 A**	20 A	13	13 A
	**6 A**	19 A	16	16 A
	**7 A**	6 A	11	11 A
C	**1 C**	16 C	16 C	7 C
	**2 C**	1 C	2 C	1 C
	**3 C**	4 C	10 C	3 C
	**4 C**	15 C	15 C	6 C
	**5 C**	10 C	1 C	5 C
	**6 C**	7 C	7 C	4 C
	**7 C**	2 C	4 C	2 C
D	**1D**	10D	11D	10D
	**2D**	12D	9D	12D
	**3D**	14D	14D	14D
	**4D**	9D	17D	9D
	**5D**	18D	21D	18D
	**6D**	21D	13D	21D
	**7D**	20D	3D	20D

^a^ The present nomenclature of chromosomes 1 A to 7D was according to the designation of oat reference assembly v1

### Integrated physical map for TR-Oligos in *A. sativa*

**Fig. 5:**
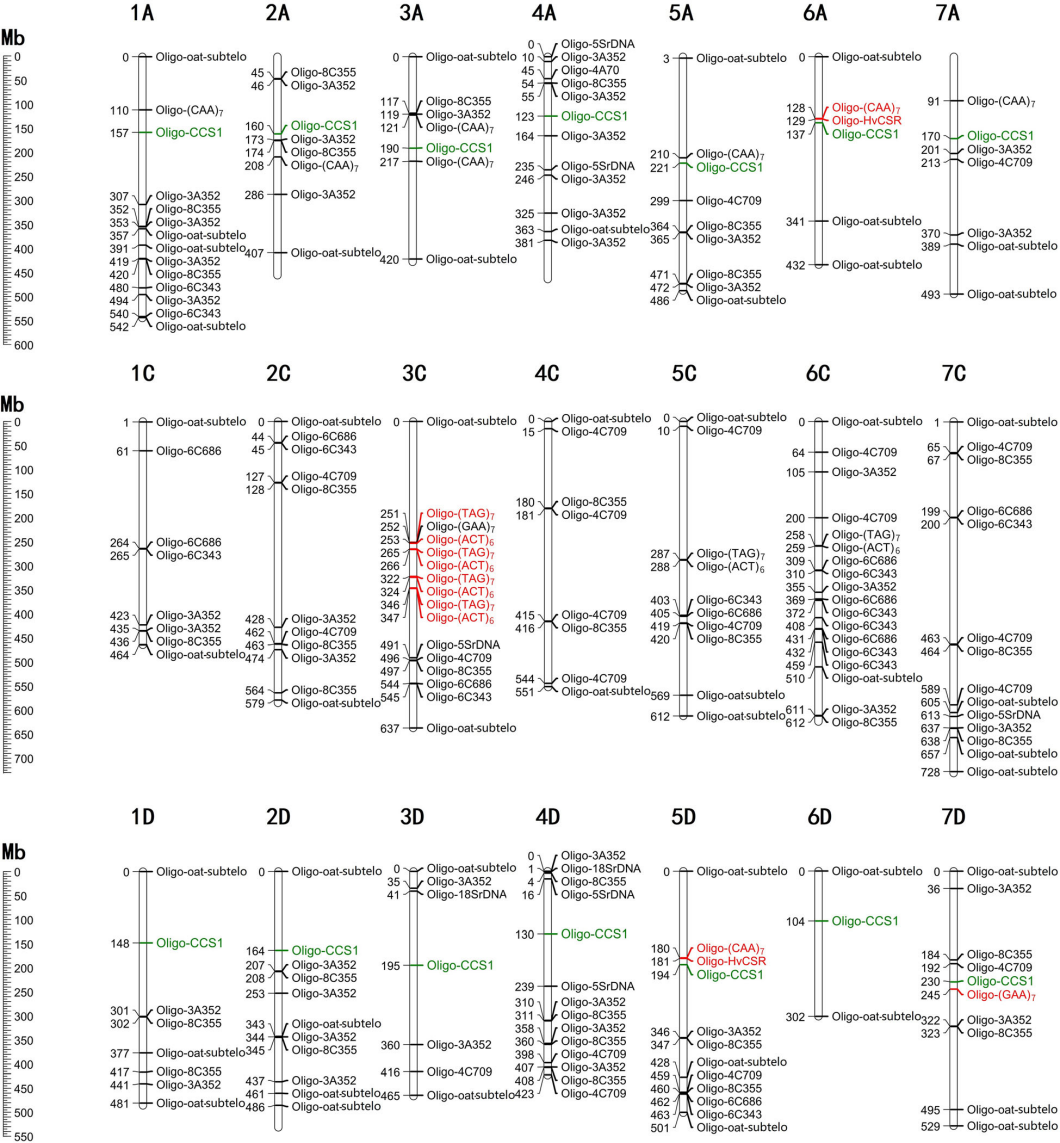
An integrated physical map of TR-oligos predicted in oat genome v1 and validated by ND-FISH. Numbers on the left represent the physical position with units of Mb. The copy number predicted over 100 is marked in red

FISH based on probes containing an SSR motif have been used for genomic evolutionary analysis [24, 43, 44]. However, the physical locations of these SSRs on the genomes of *A. sativa* were unavailable. Our established new karyotype nomenclature system based on FISH has enabled genome-wide localization of the SSRs repetitive sequences (Table 1) on common oat. The sequential ND-FISH analysis has permited localization of several repeats onto specific regions of the chromosomes of BaiyanII (Figure S3). For example, we found that Oligo-(ACT)_6_ hybridized on the pericentromeric or centromeric regions of chromosomes 3 C, 5C and 6 C, Oligo-(GAA)_7_ on chromosomes 3 C and 7D, while Oligo-(CAA)_7_ showed strong signals on 6 A and 5D, and weak signals on 1 A, 2 A, 5 A, 2D of BaiyanII (Figure S3). The FISH hybridized sites of Oligo-(GAA)_7_, Oligo-(CAA)_7_ and Oligo-(ACT)_6_ were consistent with previous reports on oat [27, 28, 43, 44]. A total of 14 non-redundant oligo-probes (Table 1) and the SSR motifs were allocated to 223 predicted chromosome locations with an accumulated copy number over 40 per 1 Mb, which relatively matches with the physical locations revealed by ND-FISH. An integrated oligo-based ND-FISH map of oat is shown in Fig. 5. The 223 hybridization sites include 66 on the A genome, 91 for the C genome and 66 in the D genome. Each chromosome appeared to have 3–19 hybridization sites, of which, the shortest chromosome 6D showed only three hybridization sites by two oligos (Fig. 5). The newly produced TR-Oligos, as well as previously reported probes (Table 1), can be used as an oligo ‘cocktail’ to detect specific chromosome regions effectively. The integrated FISH map will be extremely useful for detecting any chromosome rearrangements, as well as revealing their evolution by combined the genomic resources and cytogenetic knowledge of oat genome.

### Comparing the karyotype of *A. sativa* to wheat-barley lineage by Oligo-FISH painting

**Fig. 6:**
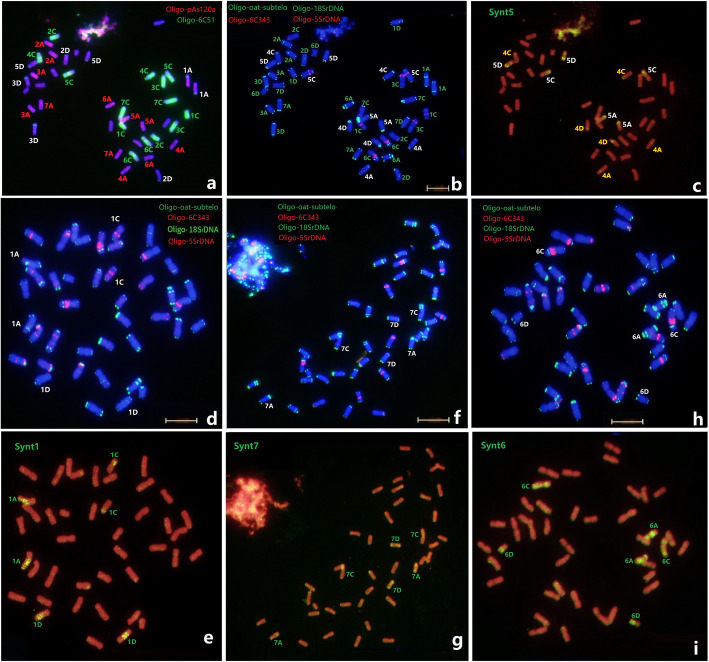
Sequential FISH of *Avena sativa* with repetitive probes and Oligo pools. The probes Oligo-pAs120a + Oligo-6C51 (**a**), Oligo-oat-subtelo + Oligo-6C343 + Oligo-18SrDNA + Oligo-5SrDNA (**b**, **d**, **f**, **h**), Synt5 (**c**), Synt1 (**e**), Synt7 (**g**), and Synt6 (**i**) were used, respectively

The standard FISH karyotype based on the single copy sequences may illustrate the low variation among genomes which has been conserved across most of evolutionary history [22]. We used the lineage-specific probes Synt1 to Synt7 representing syntenic regions between wheat and barley [37] to compare the linkage group assignment of *A. sativa* chromosomes to Triticeae species. For example, the probe Synt7 produced strong hybrid signals on the three chromosomes pairs of 7 A, 7B and 7D of wheat, and barley 7 H, as well as the linkage group 7 chromosomes of other Triticeae species [37]. Mitotic chromosomes of *A. sativa* were subjected to ND-FISH analysis using probe combinations of Oligo-6C51 + Oligo-8C355 (Fig. 6a) and Oligo-oat-subtelo + Oligo-6c343 + Oligo-18SrDNA + Oligo-5SrDNA (Fig. 6b), followed FISH by the bulked oligo probes Synt1 to Synt7 (Fig. 6). The hybridization patterns showed that Synt1 produced strong hybrid signals on the three chromosomes pairs of 1 A, 1 C and 1D of the *A. sativa* group 1 chromosomes, while Synt7 produced strong hybrid signals on the three chromosomes pairs of 7 A, 7 C and 7D of the *A. sativa* group 7 chromosomes, respectively. Similarly, the bulked painting probes Synt6 (Fig. 6), Synt 2 and Synt3 (data not shown) also mainly hybridized to corresponding linkage groups 6, 2 and 3 of oat chromosomes, respectively. We also observed that Synt4 and Synt5 hybridized to six chromosome pairs of *A. sativa* on the ends of both long and short arms, indicating that oat linkage groups 4 and 5 have undergone clear rearrangements with respect to the wheat-barley chromosome linkage groups (Fig. 6c). The results confirmed the genome synteny of the putative *Hordeum*-*Avena* orthologs by cytogenetic ND-FISH and Oligo-FISH painting, which is consistent to the prediction by Maughan et al. [35]. Therefore, our comparative bulk probe based-FISH results have demonstrated the relatively conserved collinearity of the grass genomes, and confirmed the universal karyotype system we have established as potentially useful for comparative evolutionary studies.

### The karyotype system applied for diploid *Avena* chromosome identification

**Fig. 7:**
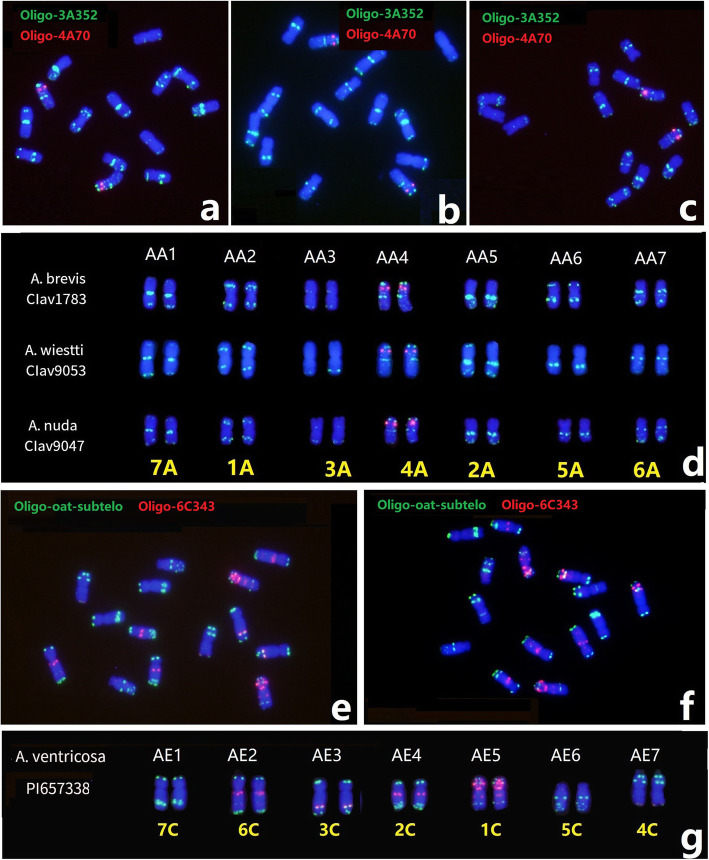
ND-FISH karyotype of diploid *Avena* species with A and C genomes by multiple oligo probes. The ND-FISH of *A. brevis* (**a**), *A. wiestti* (**b**), *A. nuda* (**c**) by Oligo-3A352 + Oligo-4A70, and *A. ventricosa* (**e**, **f**) by Oligo-oat-subtelo + Oligo-6C343, respectively. The ND-FISH karyotypes of Oligo-3A352 and Oligo-4A70 for above lines were showed with the predicted chromosomes (**d**) and (**g**), respectively

Maughan et al. [35] designated the chromosomes AA1-AA7 and AE1-AE7 for the As- and Cp-subgenomes from *A. atlantica* and *A. eriantha*, respectively. The physical locations of the probes Oligo-3A352 + Oligo-4A70 were used to study the genomes of diploid *Avena* species. ND-FISH also revealed the karyotypes of *A. brevis*, *A. wiestti* and *A. nuda* (Fig. 7d). We found that the physical distribution of the probes on AA1 to AA7 chromosomes closely matched their locations revealed by ND-FISH on the A genome of *A. sativa*. Similarly, for *A. venticosa*, the physical locations of probes Oligo-oat-subtelo + Oligo-6C343 on the C genome were consistent with the ND-FISH results on *A. sativa* (Fig. 7). Chromosomes AE5, AE4, AE3, AE7, AE6, AE2, AE1 were corresponded to the linkage groups 1-7 C, respectively (Fig. 7 g).

**Fig. 8:**
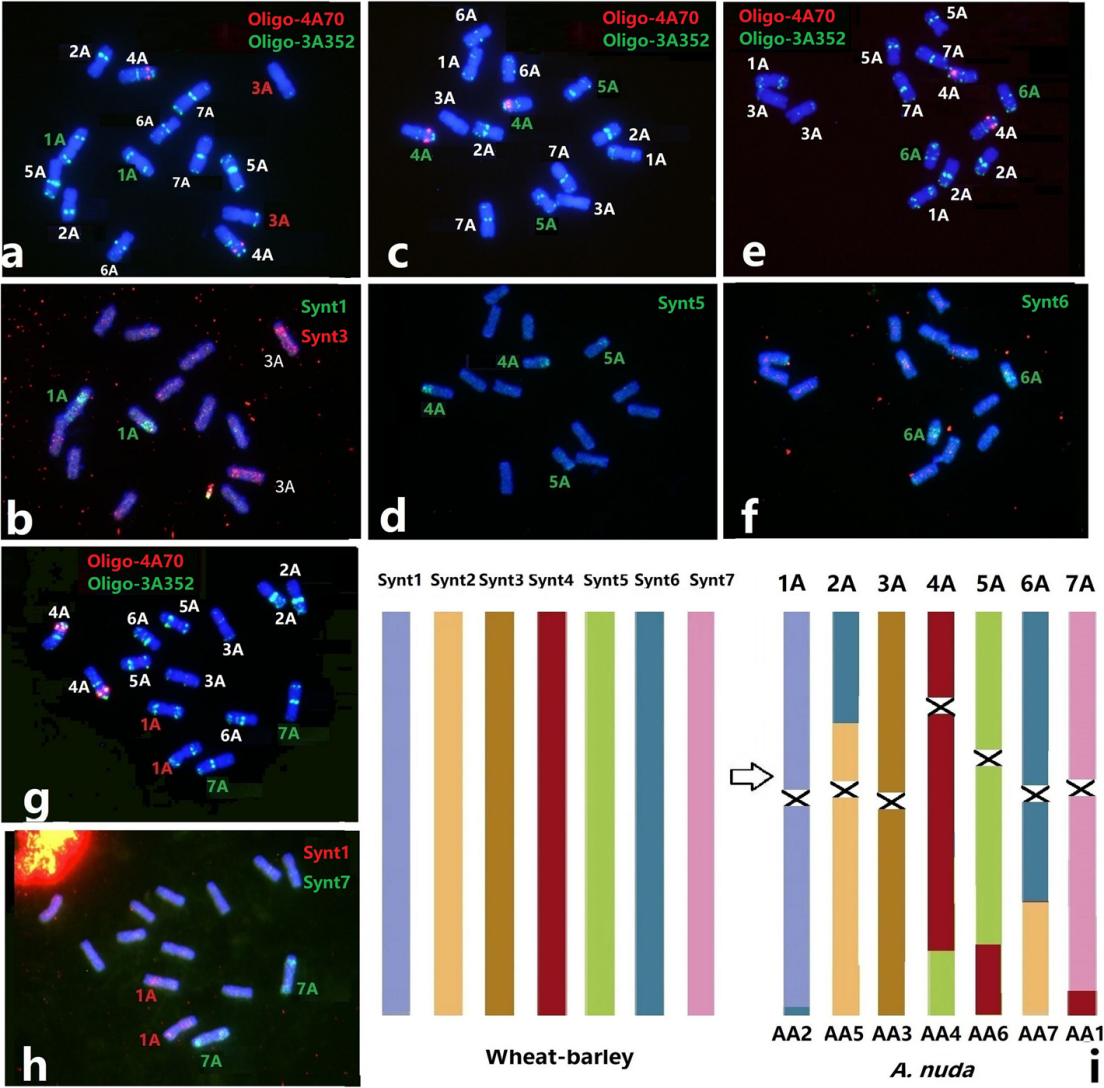
FISH of bulked oligo probes and the TR based Oligos for *A. nuda* accession CIav 1783. Hybridization with probes Oligo-4A70 + Oligo-3A352 (**a**, **c**, **e**, **g**) and subsequent stripping and re-hybridization with the bulked oligo pools Synt1 + Synt3 (**b**), Synt5 (**d**), Synt6 (**f**) and Synt1 + Synt7 (**h**) are shown. **i** A putative schematic diagram showing the A genome of diploid *Avena* compared with the common ancestor of wheat and barley

The bulked oligos Synt1 to Synt7, developed previously for FISH painting of wheat-barley chromosomes, can also be applied to the A genome chromosomes of *Avena* to reveal structural rearrangements. FISH results showed that chromosomes 1 A (AA2), 3 A (AA3) and 7 A (AA1) were structurally highly conserved (Fig. 8). Chromosomes 2 A (AA5), 4 A (AA4), 5 A (AA6) and 6 A (AA7) displayed distinct rearrangements, based on FISH with Synt1 and Synt7 probes combined with the ND-FISH by probes Oligo-4A70 and Oligo-3A352 (Fig. 8).

### Identification of chromosome rearrangements in *A. sativa*

**Fig. 9:**
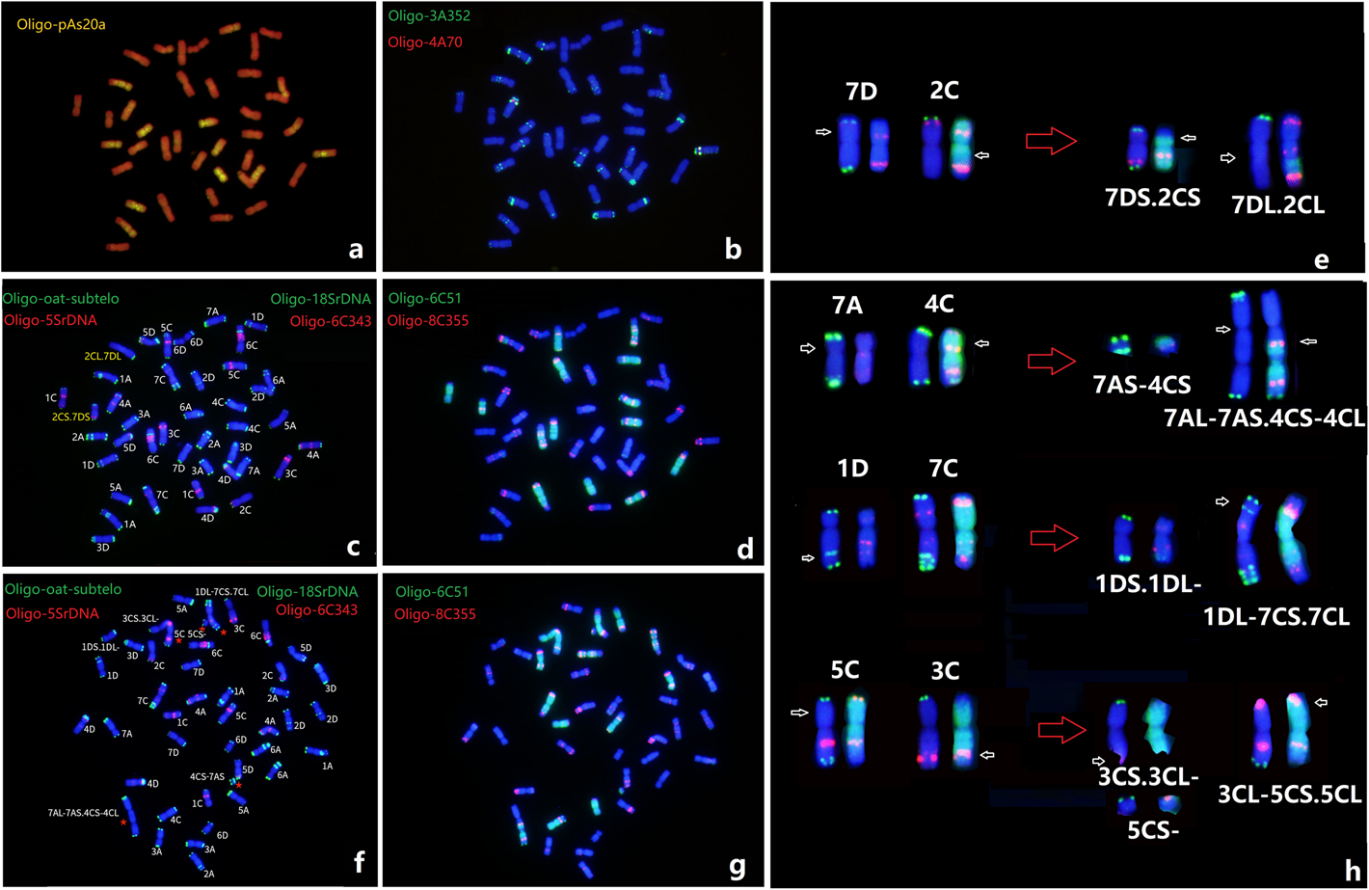
Sequential FISH karyotyping of root-tip cell from *A. sativa* lines AS112-1 (**a**-**e**) and AS112-3 (**f**-**h**) revealed multiple intergenomic translocations. The probes Oligo-pAs120 (**a**), Oligo-3A352 + Oligo-4A70 (**b**), Oligo-oat-subtelo + Oligo-6C343 + Oligo-18SrDNA + Oligo-5SrDNA (**c**, **f**), Oligo-6C51 + Oligo-8C355 (**d**, **g**), respectively. The karyotype diagrams after ND-FISH using probes Oligo-oat-subtelo + Oligo-6C343 + Oligo-18SrDNA + Oligo-5SrDNA (left), Oligo-6C51 + Oligo-8C355 (right) indicate numerous breakage points on the original oat chromosomes, as shown by arrows (**e**, **h**). Chromosome structural changes in these two lines was deduced by referring to chromosome structures of *A. sativa*

Metaphase chromosome spreads of *A. sativa* selected lines AS112-1 and AS112-3 were subjected to sequential ND-FISH using multiple oligo probes. ND-FISH revealed that line AS112-1 contained a 7D-2 C reciprocal translocation on the interstitial region of the short arm of the original 7D and long arm of 2 C chromosomes (Fig. 9a-e). Line AS112-3 contained multiple translocations involving 5C-3C, 1D-7 C and 7 A-4 C as well as dicentric and deletion chromosomes, which are identified by sequential FISH. The breakage points on the deletion, translocation, dicentric chromosomes and short midget chromosomes are shown in Fig. 9f-h. These complex chromosome rearrangements can be easily identified by several rounds of FISH with the above probes, and also with help of the established standard chromosome nomenclatures. Therefore, advanced karyotypic analysis by sequential ND-FISH on *Avena* species is effective for identifying new chromosome translocations and visualizing the precise breakpoints of the chromosomal rearrangements.

## Discussion

Standard karyotypes are generally accompanied by a universally accepted nomenclature system wherein individual chromosomes and specific regions can be numerically recognized, which provides a quick and reliable mode of discerning cross-species chromosomal or genomic similarities [21]. Traditionally, aneuploid analysis combined with individual chromosome identification has been used to assign a chromosome to a specific linkage group in diploid and polyploid plant species [22]. In hexaploid oat, the 21 distinct monosomic lines based on differences in chromosome morphology have been described [51]. Such aneuploid stocks include monosomic lines that can be difficult to maintain [6]. The development of chromosome banding techniques has long been considered as a fast, reliable, and economical method for identification of chromosomes of the *Avena* species [6, 12, 16]. However, some chromosomes or chromosome segments are lacking of diagnostic bands. Subsequently, Irigoyen et al. [15] generated a FISH map of *A. sativa* cv. SunII and its monosomic lines. They used simultaneous and sequential FISH which allowed the unequivocal identification and genome assignation of all chromosomes, including three intergenomic translocations in SunII. In light of the fact that an average *Avena* genome may contain about 76–78 % dispersed or tandem distribution of repetitive DNA sequences, Liu et al. [30] identified the nature, abundance and organization of all the repetitive DNA families in *A. sativa*, and they produced several probes suitable for use in FISH. Conventional FISH and ND-FISH methods are mostly based on the satellite repetitive sequences and are generally not linkage group-specific [7, 14, 19, 20]. Consequently, a high-resolution cytogenetic FISH map of the universal hexaploid and diploid *Avena* genome representing each of its chromosomes is essential for precise chromosome identification.

The increasing amount of available sequence data of both diploid and hexaploid oat has led to greater knowledge of the abundance and distribution of repeat-sequences across the assembled genomes of these types of species [35]. In the present study, we predicted the genome-wide tandem repeats for OT3098 genome assembly v1 by accessing data on the B2DSC web server [25], and produced seven new oligo probes which hybridized onto the chromosomes of oat (Table 1). that the TR-based Oligo probes, such as combinations of Oligo-6C51 + Oligo-8C355 and Oligo-oat-subtelo + Oligo-6C343 + Oligo-18SrDNA + Oligo-5SrDNA (Figs. 4 and 6), enable the precise and efficient identification of hexaploid oat chromosomes. The advantage of the present chromosome identification system is that it employs sequential ND-FISH with an increased number of probes which exhibit clear locations. Our procedure will improve the reliability for chromosome identification (Fig. 5), and the resolution can thus be increased by the ongoing updated version of oat genome assembly. We have also investigated the degree of repetitive DNA composition in genomes from the wild relatives of oat by ND-FISH (Fig. 7). Future research will extend this study to a number of other diploid and tetraploid species for quantifying the magnitude of intra- and interspecific variation.

The time periods for divergence between oats and members of the Triticeae (wheat, barley) has been estimated to be 25.5– 26.5 MYA between oats and wheat, and 23–25 MYA between oats and barley [40]. Whole-genome comparisons with barley and wheat have revealed that extensive blocks of synteny remain which have helped resolve homologous relationships between different oat linkage groups [32]. The wheat-barley genome sequences will be useful resources to assist genetic and genomic researches in oat [30, 35]. Based on the comparative FISH between diploid and hexaploid oat by Oligo-FISH painting with probes Synt1 to Synt7 (Fig. 6), we found that the comparative maps may help to resolve homologous relationships between different linkage groups and reveal many undiscovered major rearrangements in *Avena* subgenomes. Moreover, the estimated time of divergence of the two more similar subgenomes (A/D) from the distinct one (C) was around 7.9–8.7 MYA [40]. It also possibly suggests that large chromosome structural rearrangements may have occurred between diploid and hexaploid *Avena* species, as revealed by genome-wide comparisons between the As and Cv genomes of diploid species to those of A and C genomes of the hexaploid oat (Figure S4). The comparative bulked probes based FISH results showed the overall relatively conserved genome collinearity of the *Avena* species across the different ploidy levels (Figs. 6 and 8). Our results demonstrated that the development of region-specific Oligo-FISH probes based on oat sequences is useful to identify individual homoeologous chromosomes from distantly *Avena* species. The present comparative oligo-based FISH studies and later development of more dense landmarks will provide new insights into the evolution of *Avena* genera.

In addition, the lack of a high-density marker system has limited the application of genomic selection in cultivated oat. The accuracy of genomic selection has continually increased since the linkage maps have been improved by genetic, cytogenetic and genomic advances [52]. Chaffin et al. [18] published a map representing the most common physical chromosome arrangements in oat. Deviations from the consensus map may indicate physical rearrangements and large chromosomal translocations may vary among different varieties. The present system has enabled precise definition of breakpoints of translocation chromosomes (Fig. 9), which has great potential for the high-throughput karyotyping of the chromosome structure for evolutionary diverged genomes. Some FISH probes specifically hybridized to oat and produced no hybridization signals in wheat and other grass species. Therefore, our FISH protocol may have additional applications in tracing *Avena* chromatin introgressed into wheat or maize [46, 53, 54] following wide crosses and chromosome manipulation based breeding practices.

## 4. Methods

### Plant materials

The CIav and PI accessions of wild and cultivated *Avena* accessions were provided by United States Department of Agriculture, Agricultural Research Service. The cultivated oat lines BaiyanII and AAC Nicolas were maintained in Shanxi Agricultural University, China. Seeds of natural accessions of hexaploid oat lines AS111 and AS112 were maintained in the Laboratory of Molecular and Cell Biology, Center for Informational Biology, School of Life Science and Technology, University of Electronic Science and Technology of China in Chengdu. The materials and their chromosome constitution are listed in Table 3.

**Table 3:** Materials and their genome constitution used in this research

No	Taxa	Voucher (Repository)	Origin country	2n	Genome(s)
1	* A. brevis* Roth	CIav 1783 (NSGC)	Niedersachsen, Germany	14	As
2	*A. nuda* L.	CIav 9047 (NSGC)	England, United Kingdom	14	As
3	*A. wiestii* Steud.	CIav 9053 (NSGC)	Ontario, Canada	14	As
4	*A. ventricosa* Balansa ex Coss.	PI 657,338 (NSGC)	Morocco	14	Cv
5	*A. eriantha* Durieu	PI367381 (NSGC)	Madrid, Spain	14	Cp
6	*A. fatua* L.	CIav 2527 (NSGC)	Alberta, Canada	42	ACD
7	*A. sativa* L.	Baiyan II (SXAU)	Jilin, China	42	ACD
8	*A. sativa* L.	AAC Nicolas (ORDC)	Ottawa, Canada	42	ACD
9	*A. sativa* L.	CIav 3520 (NSGC)	Germany	42	ACD
10	*A. sativa* L.	AS111 (UESTC)	Shanxi, China	42	ACD
11	*A. sativa* L.	AS112 (UESTC)	Shanxi, China	42	ACD

Voucher: PI or CIav, Germplasm Resources Information Network of United States Department of Agriculture at Beltsville, USA; NSGC, National Small Grains Collection, USA; SXAU, Shanxi Agricultural University, China; ORDC, Ottawa Research and Development Centre, Canada; UESTC, University of Electronic Science and Technology of China. Genome assignment based on Liu et al. [8]

### Bioinformatic analysis of repeats in *Avena* genome

The genome sequences, including diploid oat [35] and cultivated oat OT3098 reference assembly v1 (https://wheat.pw.usda.gov/GG3/graingenes_downloads/oat-ot3098-pepsico) were download for prediction of tandem repeats using the TRF software [55]. The physical locations of tandem repeats was according to the B2DSC method with default parameters (http://mcgb.uestc.cn/b2dsc) described by Lang et al. [25]. Thirteen novel oligo probes with physical location in *Avena* genomes for ND-FISH were designed and listed in Table 1.

### Chromosome preparation, Sequential C-banding and FISH analysis

Root tips from germinated seeds were collected and treated with nitrous oxide followed by enzyme digestion [56]. The Giemsa C-banding was done according to according to Li et al. [57]. The TR based probes with the synthetic oligos were labeled with either 5’ end-labelled 6-carboxyfluorescein (6-Fam) for green or 6-carboxytetramethylrhodamine (Tamra) for red signals. The protocol of non-denaturing FISH (ND-FISH) using synthesized probes was described by Fu et al. [58]. The wheat-barley linkage group specific bulked oligo pool probes (Synt1 to Synt 7) were designed following our recently published procedure [37]. After oligo-based FISH, sequential FISH painting with oligo pool probes was conducted according to Han et al. [38] and Bi et al. [59]. Photomicrographs were taken with an Olympus BX-53 microscope equipped with a DP-70 CCD camera.

## Conclusions

The availability of the Oligo-based FISH system opens the way in the genus *Avena* for comprehensive cytogenetic analysis combined with genomics tools. We have demonstrated that ND-FISH with a new set of tandem repeat probes, combined with FISH painting by oligo pools, can generate a high resolution and informative cytogenetic map on genome regions for the cultivated oat. The consensus karyotype based on ND-FISH, by physical mapping of labeled probes, can effectively substitute for traditional cytological methods in *Avena* for identifying genomic rearrangements. Our current cytogenetic mapping efforts, integrated with genomic approaches, will provide a new perspective to address important questions involving chromosome evolution in *Avena* species, as well as wide-cross and chromosome manipulation-based breeding in oat.

## Supplementary Information

**Additional file 1:** **Table S1.** The predicted total TRs in the chromosomes of oat genome assembly v1 by TRF., **Table S2.** Distribution and copy number prediction of centromeric repeats Oligo-CCS1 in oat genome assembly v1., **Figure S1.** The genomic distribution of TRs in assembled oat genome v1 revealed by each chromosome., **Figure S2.** ND-FISH karyotype of A. sativa revealed by multiple oligo probes of tandem repeats., **Figure S3.** Sequential ND-FISH analysis by predicted TR Oligo-probes on metaphase chromosomes of oat BaiyanII., **Figure S4.** The comparative genome between the A-genome of A. sativa to A-genome of A. atlantica (a), C-genome of A. sativa to AE genome of A. eriantha (b) by Circos software with the annotated genes.

## Data Availability

The data that support the findings of this study are included in this published article and its additional files.
