# Supplementary material for: A universal karyotypic system for hexaploid and diploid Avena species brings oat cytogenetics into the genomics era
Source: BMC Plant Biol. 2021 May 12;21:213. doi: 10.1186/s12870-021-02999-3 (PMC8114715; doi:10.1186/s12870-021-02999-3)
Supplement: Supplementary file 1 — Additional file 1: Table S1. The predicted total TRs in the chromosomes of oat genome assembly v1 by TRF., Table S2. Distribution and copy number prediction of centromeric repeats Oligo-CCS1 in oat genome assembly v1., Figure S1. The genomic distribution of TRs in assembled oat genome v1 revealed by each chromosome., Figure S2. ND-FISH karyotype of A. sativa revealed by multiple oligo probes of tandem repeats., Figure S3. Sequential ND-FISH analysis by predicted TR Oligo-probes on metaphase chromosomes of oat BaiyanII., Figure S4. The comparative genome between the A-genome of A. sativa to A-genome of A. atlantica (a), C-genome of A. sativa to AE genome of A. eriantha (b) by Circos software with the annotated genes. [file 12870_2021_2999_MOESM1_ESM.docx]

**Suplementary Information**

A universal karyotypic system for hexaploid and diploid *Avena* species brings oat cytogenetics into the genomics era

Wenxi Jiang^1^, Chengzhi Jiang^1^, Weiguang Yuan^1^, Meijun Zhang^2^, Zijie Fang^1^, Yang Li^1^, Guangrong Li^1^, Juqing Jia^2,^* and Zujun Yang ^1,^*

^1^Center for Informational Biology, School of Life Science and Technology, University of Electronic and Technology of China, Chengdu 611731, China

^2^College of Agronomy, Shanxi Agricultural University, Taigu 030801, China

*Corresponding authors:

Juqing Jia, E-mail: [jiajuqing@126.com](mailto:jiajuqing@126.com)

Zujun Yang, E-mail: [yangzujun@uestc.edu.cn](mailto:yangzujun@uestc.edu.cn)

**Additional files**

**Table S1.** The predicted total TRs in the chromosomes of oat genome assembly v1 by TRF.

| chromosome | TR total length (bp) | Chromosome Length (bp) | TR content (%) |
| --- | --- | --- | --- |
| 1A | 13716046 | 542795238 | 2.53 |
| 2A | 12331120 | 454026946 | 2.72 |
| 3A | 15077311 | 426317889 | 3.54 |
| 4A | 17454436 | 462057589 | 3.78 |
| 5A | 12271647 | 485535456 | 2.53 |
| 6A | 6297372 | 431567647 | 1.46 |
| 7A | 8190218 | 493489733 | 1.66 |
| 1C | 24229298 | 463431985 | 5.23 |
| 2C | 40130565 | 585391692 | 6.86 |
| 3C | 45492946 | 636099650 | 7.15 |
| 4C | 38144143 | 552251759 | 6.91 |
| 5C | 39100967 | 612252875 | 6.39 |
| 6C | 54168228 | 624915216 | 8.67 |
| 7C | 49889994 | 731989224 | 6.82 |
| 1D | 11055156 | 485732902 | 2.28 |
| 2D | 13662316 | 532459853 | 2.57 |
| 3D | 6727829 | 467934025 | 1.44 |
| 4D | 13884029 | 424978419 | 3.27 |
| 5D | 12012596 | 502323219 | 2.39 |
| 6D | 3349097 | 301592285 | 1.11 |
| 7D | 10026839 | 529301501 | 1.89 |

**Table S2**. Distribution and copy number prediction of Centromeric repeats Oligo-CCS1 in oat genome assembly v1

| Chromosome | Position (Mb) | Copy number |  |
| --- | --- | --- | --- |
| 1A | 150-160 | 522 |  |
| 2A | 149-162 | 620 |  |
| 3A | 183-193 | 497 |  |
| 4A | 113-128 | 692 |  |
| 5A | 211-226 | 894 |  |
| 6A | 130-144 | 686 |  |
| 7A | 161-174 | 743 |  |
| 1D | 142-152 | 558 |  |
| 2D | 154-169 | 647 |  |
| 3D | 181-196 | 573 |  |
| 4D | 111-132 | 619 |  |
| 5D | 176-196 | 625 |  |
| 6D | 95-106 | 555 |  |
| 7D | 214-233 | 747 |  |


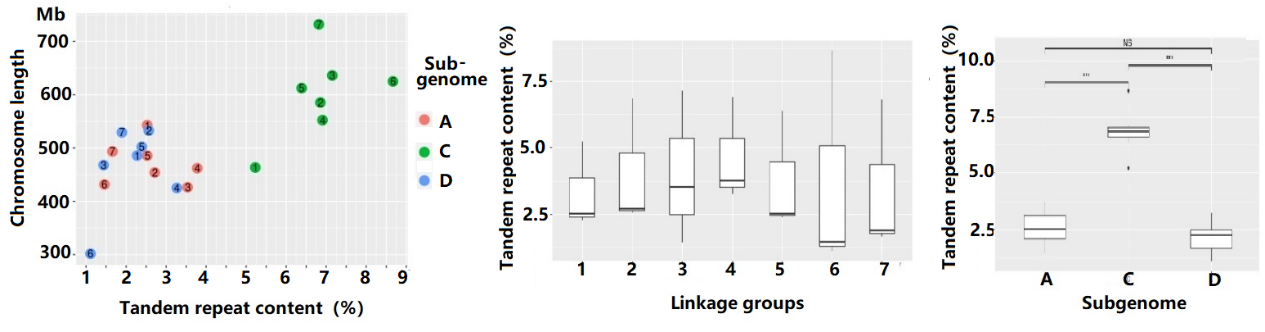


**Fig. S1.** The genomic distribution of TRs in assembled oat genome revealed by each chromosome (a), linkage group (b) and sub-genomes (c).


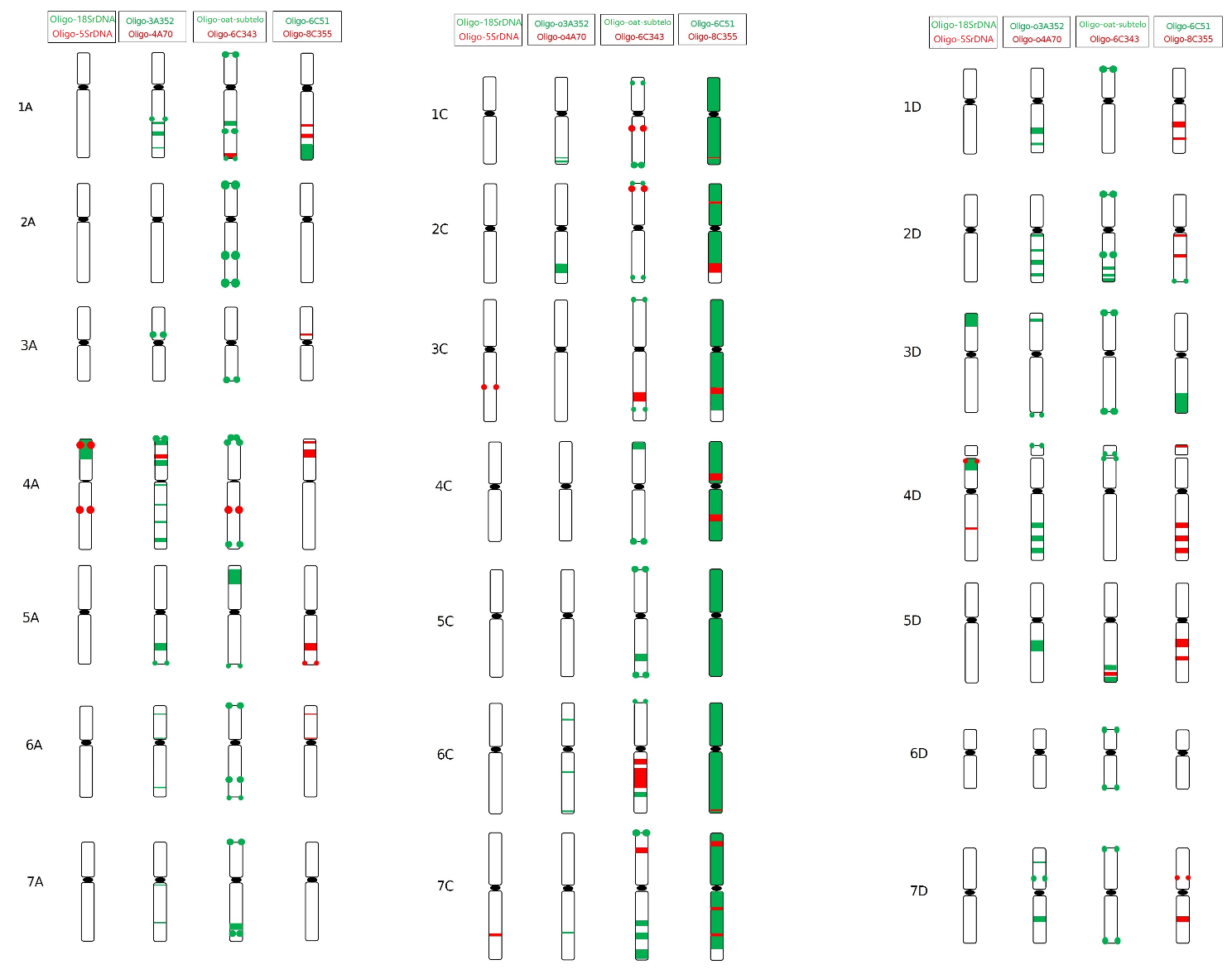


**Fig. S2**. ND-FISH karyotype of *A. sativa* revealed by multiple oligo probes of tandem repeats


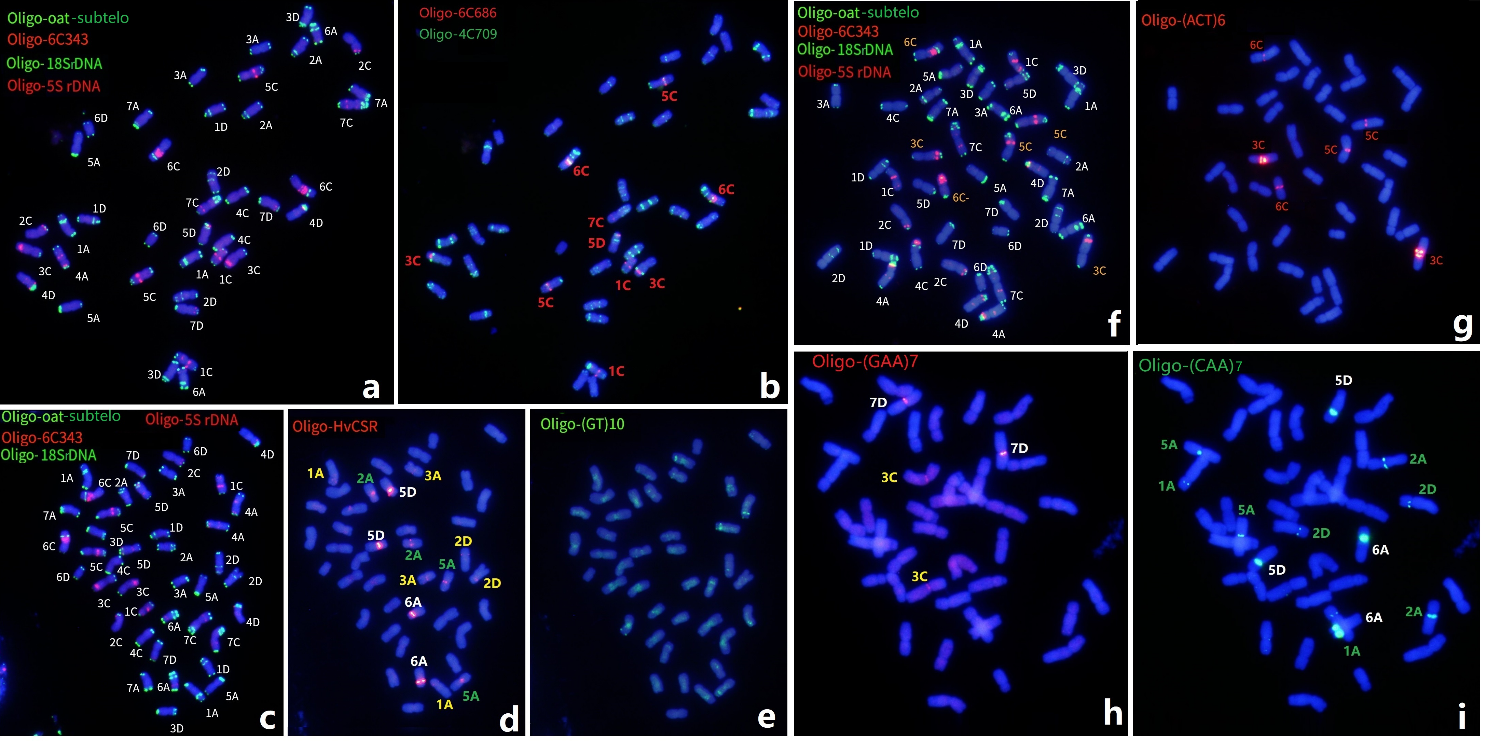


**Fig. S3.** Sequential ND-FISH with tandem repeat (TR) Oligo-probes onto metaphase chromosomes of oat cv. BaiyanII.

The probes used are: Oligo-oat-subtelo + Oligo-6C343 + Oligo-18SrDNA + Oligo-5SrDNA (**a, c, f**), Oligo-6C686 + Oligo-4C709 (**b**), Oligo-HvCSR (**d**), SSR motifs of Oligo-(GT)_10_ (**e**), Oligo-(ACT)_6_ (**g**), Oligo-(GAA)_7_ (**h**), and Oligo-(CAA)_7_ (**i**). The chromosomal locations of the hybridized probes are marked on each figure.


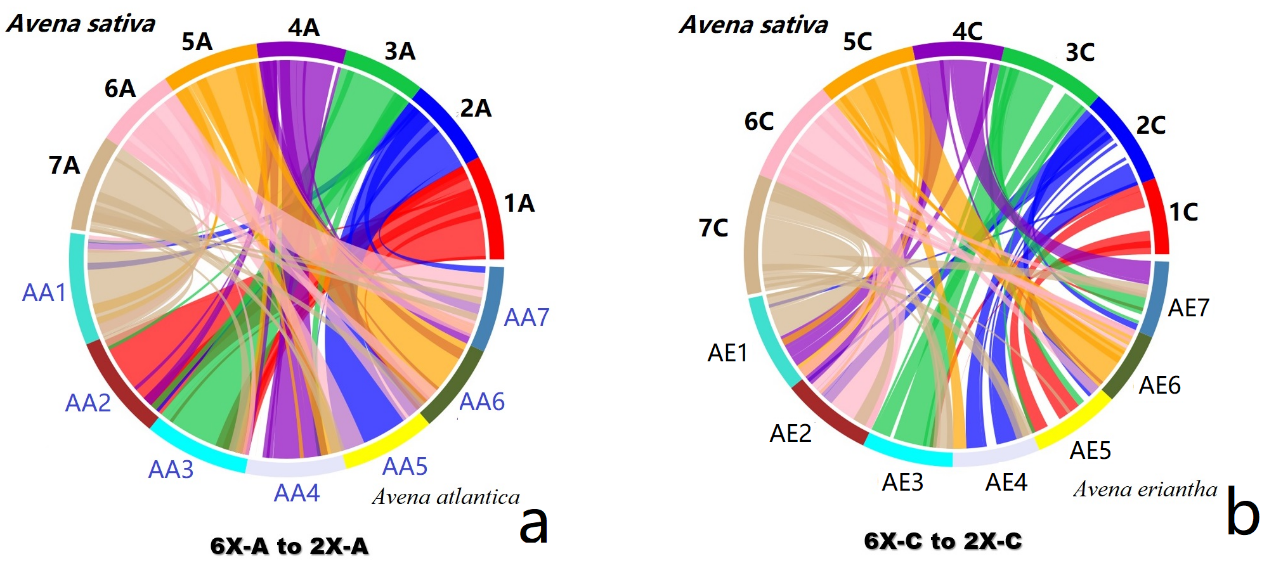


**Fig. S4**. The comparison between the *A. sativa* A-genome to *A. atlantica* AA-genome (a), and the *A. eriantha* AE genome (b), by Circos software with the annotated genes.
